# An assessment of the impacts of child oral health in Indonesia and associations with self-esteem, school performance and perceived employability

**DOI:** 10.1186/s12903-017-0358-5

**Published:** 2017-03-21

**Authors:** Diah Ayu Maharani, Melissa Adiatman, Anton Rahardjo, Girvan Burnside, Cynthia Pine

**Affiliations:** 1 Department of Preventive and Public Health Dentistry, Faculty of Dentistry, Universitas Indonesia, Jalan Salemba No. 4, Jakarta, 10430 Indonesia; 2 Department of Biostatistics, University of Liverpool, Liverpool, England, UK; 3 Dental Public Health, Barts and The London Institute of Dentistry, Queen Mary University of London, Whitechapel, London, England, UK

**Keywords:** Caries, Children, Indonesia, Oral health, Self-esteem

## Abstract

**Background:**

Previous surveys have indicated that a majority of Indonesian children have poor oral health. However, little detailed information is available on underlying causation and none that examine impacts of oral health on child self-esteem, school performance and perceived employability. The aim of this study was to determine levels of child oral health in primary school children in Indonesia, the prevalence of key causal factors; and, to determine relationships between oral health, self-esteem and school academic performance.

**Methods:**

Cross-sectional epidemiological study in a sample (*n* = 984) of children aged 6–7 and 10–11 years old attending three public schools in Indonesia. A dental visual impact study was conducted, in which teachers reported their perceptions of the impact of child oral health on school academic performance. Oral health behaviors, self-esteem, and school performance were assessed. The children were clinically examined to measure dental caries and oral cleanliness.

**Results:**

Teachers believe that children with visually poor oral health and impaired smiles are more likely to perform poorly at school, be socially excluded and have lower job prospects than their peers with visually good oral health and healthy smiles. The percentages of children with decayed teeth were 94 and 90% in the 6-7- and 10–11-year age groups, respectively. Families reported high levels of child consumption of sugar-containing foods and drinks; many had irregular use of fluoride toothpaste. Children with substantial plaque on their teeth achieved significantly lower levels of school performance than their peers with clean teeth. Significant associations were found between school performance and self-esteem for these children.

**Conclusions:**

The study findings highlight the need for preventive care programs to improve the oral health of children in Indonesia and prospective determination of associations between child oral health; self-esteem and school academic performance.

**Electronic supplementary material:**

The online version of this article (doi:10.1186/s12903-017-0358-5) contains supplementary material, which is available to authorized users.

## Background

Indonesia faces significant challenges in relation to poor oral health in children; this problem continues into teenage and adult years, when more than 70% are affected by experiences related to dental caries [1]. Previous studies have shown that inequality in dental care persists in Indonesia [2, 3]. Undoubtedly, broader social determinants of health have a major impact on expressed oral health and life opportunities [4]. The recent Bali Declaration, expressed at the 7th Asian Conference of Oral Health Promotion for School Children, declared that oral diseases, particularly dental caries, are major public health problems in the Asian region and that the burden of disease in children causes significant negative impacts on their health in terms of growth, and social and emotional well-being [5]. Untreated dental caries in children causes toothache, generalized pain and oral sepsis, leading to reduced food choices, lost days from school for children and lost time from work for parents. In other countries, a clear association has been shown between poor dental health in children and school performance, affecting their future employment prospects [6, 7]. No such data have been available regarding Indonesia. The critical role played by the frequent consumption of sugared foods and drinks in caries development has been recognized. Dental experts need to work closely with other professionals to support families to decrease child caries prevalence by reducing sugar intake. Brushing teeth twice daily with fluoride-containing toothpaste can adjust the oral balance towards re-mineralization [8].

Recent studies of the oral health of Indonesian children have provided data on caries prevalence and on tooth-brushing frequency [9, 10]; however, little is known about the relationships between child oral health, oral health-related behaviors, child self-esteem, child school performance and oral health parameters. Studies in other countries indicate that poor oral health can lead to social exclusion and hinders future employment, thereby reducing a child’s ability to succeed in life [6]. Whether the visual appearance of poor oral health has a similar impact in Indonesia is not known. Improving child oral health and cleanliness by reducing sugar consumption and regular tooth brushing with fluoride toothpaste might positively affect development, improving a child’s likelihood of engaging in learning at school. In preparation for evaluating a school-to-home intervention to enhance the oral health of children, baseline measures were conducted to test the following hypotheses.

1. Teachers associate children’s oral health and smile appearance with predicted school performance and job prospects.
2. Presence of dental caries in Indonesian children is associated with reported toothbrushing habits, reported sugar consumption, and observed oral cleanliness (plaque and gingivitis).
3. Primary school children’s school performance is associated with the following variables; reported tooth brushing habits, observed oral cleanliness (plaque), dental caries experience, school absentee rate, and children’s self-esteem.
4. Dental caries experience and their sequelae e.g. toothache are associated with primary school children’s self-esteem

## Methods

### Ethics committee and study approvals

The study protocol was considered and approved by the Ethics Committee of the Faculty of Dentistry, Universitas Indonesia (reference number 91/XI/13). Other approvals were obtained from the Department of Health and Head Teachers of the selected schools.

### Population

The population for this study included children attending three public elementary schools (Jatibening IV, Kayuringin XIII and Kayuringin XXIII) in Bekasi, a city in West Java, on the eastern border of Jakarta, the capital city of Indonesia. Bekasi is one of the Indonesia's most populated suburbs and serves as a commuter city for Jakarta; although it has notable trade, business and processing industries, substantial urban areas surround the city. These three schools are typical of the Indonesian public elementary school system in terms of size, infrastructure and systems, receiving full support and provisions from the Government. All three schools are similar in terms of location and socio-demographics and are attended only by children aged 6–12 years. The socio-demographic profile of the children attending these schools and their families are similar, representing the second lowest socio-economic quintile of the Indonesian population. The schools were chosen in partnership with the Education Department as being representative of public schools in the area and willingly took part in the study.

### Sample

All parents with children aged 6–7 years (grades 1 and 2) and those with children aged 10–11 years (grades 5 and 6) were invited to join the study and were provided with a participant information leaflet and a consent form. Purposive sampling was used to ensure that the samples were representative of the majority school children in Indonesia who are from the second lowest socio-economic quintile. Sampling involves the selection of a number of study units from a defined study population [11]. Unfortunately, few research articles on dental health in Indonesia have been published [2]. Therefore, many of the parameters to be explored within this population were unknown, thus limiting the ability to undertake an accurate formal sample size calculation. In these circumstances WHO Oral Health Surveys Basic Survey Methods (WHO, 2013) [12] advises a sample size of 25–50 children per age group. Parameters in addition to oral health were to be assessed, therefore, to obtain sufficient data for comparison and hypothesis testing, a sample size of at least 400 children aged 6–7 years and 400 children aged 10–11 years was estimated [13]. The samples were to be selected from a minimum of twenty classes (clusters). This sampling methodology was approved by the Ethical Committee. All children in the two age groups in all three schools were included in the sampling frame. The inclusion criteria were children for whom a parent or guardian had given consent to participate in the study and agreed to complete the questionnaires.

### Dental visual impact study: Teachers’ assessment of the impact of child oral health on school performance

A Teachers’ Workshop was conducted by a local dental expert from the University of Indonesia (AR). The teachers are Government Employee who live in Bekasi city and represent the middle class of the population. Teachers viewed a short series of slides showing children’s smiles. After viewing each slide, each teacher was asked to complete a form (see Additional file 1). This gave their judgments of the child’s likely health status, school performance and employment prospects. Each slide was standardized by showing only the smile of the child (not the rest of their appearance), thus excluding the possibility that the teachers might be influenced by other factors e.g., general attractiveness and other facial features. Moreover, the slides did not show any “ugly duckling stage” of the mixed dentition phase. The slides showed either healthy smiles (teeth without visible decay) or impaired smiles caused by decayed upper primary teeth only. Children represented the same broad ethnic population as the teachers (Indonesian family backgrounds); thus, lip shapes and skin color were familiar. No information was provided regarding the child’s academic record or their health. The children were all of primary school age, approximately 6 to 11 years and they were not pupils in the schools where the teachers were employed. All teachers in the selected schools were invited to a workshop, and the dental visual impact study was part of the workshop. This explorative study sought to assess teachers’ perceptions of the impact of children’s oral health, as judged by their smile appearance, on children’s predicted school performance and job prospects. Therefore, the dental visual impact study was a pilot study, with a convenience sample size of 30 participants. For descriptive studies, this sample size was large enough to reflect important variations in the study population, but small enough to facilitate the study [14]. Face and content validity were assessed through panel discussions between experts (academics and clinicians working in the field), as well as through a small scale pilot study of a sample drawn from the target population. These procedures explored the comprehensiveness, relevance and understanding of the content of the dental visual impact study form [15].

### Measures of oral health behavior, self-esteem, and child's school performance

Children aged 6–7 years and 10–11 years were asked to complete a specially developed simple measure describing their hygiene habits both general (hand and face washing) and oral (such as tooth brushing) as a self-report of their daily hygiene routines (see Additional file 2). This questionnaire had been previously developed and piloted in children in a multi-cultural community in the UK (Pine, personal communication, 2016). The simple questionnaire was found to be easily understood and completed by children in the schools in Indonesia. Children aged 10–11 years were asked to complete a self-esteem measure, (see Additional file 3) including sub-scales of peer and school connectedness [16, 17].

Parents were asked to complete the oral health behaviors questionnaire (see Additional file 4), which was developed as part of an international study of childhood caries [18]. The questionnaire was developed within an international consortium to analyze familial and cultural perceptions and beliefs of oral hygiene and dietary practices. The oral health behaviors questionnaire was developed with parents from a wide range of countries, of diverse ethnic groups, from deprived and non-deprived backgrounds and for those with children who had experienced caries and those who had not [18]. This questionnaire reports on child and parental oral health behaviors, including dental attendance and children’s experience of toothache. It measures parental self-efficacy in relation to child tooth brushing and the control of child dietary sugar; and, oral health-related beliefs and attitudes. The questionnaire was developed in English. Translation into the Indonesian language was conducted by a native speaker, pre-tested and then back translated by another native speaker to ensure comparability to the original form. The school performance of the children was measured objectively based on the results of recent tests in mathematics [19] (range of scores 0–100), from which the following categories were defined: Underachieving (0–54), Fair (55–64), Good (65–79), and Excellent (80–100).

### Clinical examinations

All children for whom parental consent had been obtained and who were present in school received a clinical examination of their oral health by one of two clinical examiners who were trained and experienced in standardized clinical survey measurements. The criteria used for the diagnosis of dental caries are those described by the World Health Organization [12]; very early enamel-only caries is not scored; however, caries that included an unmistakable cavity, undermined enamel, or detectably softened floors or walls was scored. Child oral cleanliness was measured by plaque assessments at dental examinations using a dental probe [20, 21]. For all children, the buccal surfaces of index teeth were examined. Index teeth for those aged 10–11 years were all first permanent molars and the upper left and lower right lateral incisors. For children aged 6–7 years, index teeth were upper and lower incisors and upper right molars and lower left molars. This measure was categorized into no visible plaque deposits, substantial plaque visible, and abundant bleeding on gentle probing.

### Statistical analysis

Descriptive statistics were conducted to analyze the visual impact study, demographic profile of the children and families, child oral health, and parental report of child oral health-related behaviors. The chi square test and ANOVA was employed for categorical variables and comparisons of variables related to oral health respectively (significance level: *p* < 0.05).

## Results

### Sample

The study included 984 children, of whom 539 were aged 6–7 years and 445 were aged 10–11 years. The children were equally distributed between boys and girls, as shown in Table 1. All (100%) families invited to take part in the study agreed to do so. Mothers’ educational level and parents’ self-reports on oral health and related behavior are also described in Table 1. Thirty teachers in the three schools participated in the dental visual impact study.

**Table 1:** Demographic profile of the children and families, child oral health, and parental report of child oral health-related behaviors

	6–7 years old	10–11 years old
Child gender	*n* = 539	*n* = 445
Male	245 (46%)	188 (42%)
Female	255 (47%)	201 (45%)
Missing gender data (no parent questionnaire)	39 (7%)	56 (13%)
Decayed teeth		
Mean number of decayed primary teeth (SD)	7.8 (4.4)	1.4 (2.0)
Mean number of decayed permanent teeth (SD)	0.8 (1.2)	2.7 (2.4)
Percentage of children with no decayed teeth		
% dt = 0	31 (6.4%)	207 (51.8%)
% DT = 0	301 (62.5%)	73 (18.3%)
% dt and % DT = 0	29 (6.0%)	41 (10.3%)
Child oral cleanliness		
Substantial plaque visible	238 (49.4%)	153 (38.3%)
Abundant bleeding on gentle probing	63 (13.1%)	68 (17.0%)
Missing data	57 (10.6%)	45 (10.1%)
Mothers’ education level		
Primary education	107 (20%)	68 (15%)
Secondary education	217 (40%)	152 (34%)
Higher education	95 (18%)	68 (15%)
Missing data	120 (22%)	157 (35%)
Parent report of their own tooth-brushing behavior		
Less than twice daily	77 (24%)	49 (22%)
Twice daily or more	187 (59%)	126 (56%)
Missing data	53 (17%)	51 (23%)
Parent report of their child’s tooth-brushing behavior		
Never	7 (1.6%)	3 (1.0%)
Not every day/every other day	20 (4.6%)	13 (4.2%)
Once a day	23 (5.3%)	16 (5.2%)
Twice a day	338 (77.7%)	230 (74.9%)
Three times a day	47 (10.8%)	45 (14.7%)
Missing data	104 (19.3%)	138 (31.0%)
Parent report of their child’s sweet-eating frequency		
Every day	90 (21.1%)	42 (14.1%)
Most days	102 (23.9%)	60 (20.1%)
Once a week/occasionally	234 (43.4%)	195 (43.8%)
Never	1 (0.2%)	2 (0.7%)
Missing data	112 (20.1%)	146 (32.8%)
Parent report describing how they sweeten their child’s drinks		
Sweeten with sugar	187 (44.2%)	149 (51.0%)
Sweeten with honey	66 (15.6%)	28 (9.6%)
Sweeten with condensed milk	62 (14.7%)	39 (13.4%)
Never sweeten drinks	115 (27.2%)	77 (26.4%)
Missing data	116 (21.5%)	153 (34.4%)
Parent report of what drinks are sweetened		
Sweeten milk	131 (31.0%)	70 (24.1%)
Sweeten water	9 (2.1%)	6 (2.1%)
Sweeten tea	260 (61.6%)	196 (67.4%)
Missing data	117 (21.7%)	154 (34.6%)

### Dental visual impact study

The dental visual impact study measured teachers’ perception, of whether a child was healthy and their likely future school performance and employment prospects based on slides of individual children smiling showing their teeth. Of the 30 teachers who participated in the dental visual impact study, 29 (97%) of those viewing a slide showing a child’s smile with caries-free teeth judged that the child was healthy, and 26 (87%) of those viewing a slide showing a child’s smile with decayed upper primary teeth judged that the child was unhealthy. A child with a healthy smile was judged by 21 (70%) of teachers to be more likely to do well academically; the opposite was the case for a child showing teeth that were decayed, with 20 (67%) of teachers judging such a child as less likely to do well academically in the future. Similarly, 21 (70%) of teachers judged a child presenting a healthy, caries-free smile to be more likely to have good employment prospects; only 5 (17%) of teachers judged a child with their front teeth affected by tooth decay to be more likely to have good employment prospects. In fact, a child exhibiting poor oral health was judged by 12 (40%) of teachers to be less likely to have good employment prospects; 8 (27%) of teachers were uncertain regarding the future employment prospects of a child with an unhealthy smile; but only 10% of teachers were uncertain regarding the future employment prospects of a child with a healthy dental appearance.

### Parent and child-completed questionnaires and dental examinations of children

Data from dental examinations was available for 482 (89%) of the children aged 6–7 years and for 400 (90%) of the children aged 10–11 years. The remaining children were absent from school on the day of the clinical examinations. Not all parents completed the questionnaires; for example, in relation to children’s tooth brushing habits, data were available for 331 (61%) of the children aged 6–7 years and 307 (69%) of the children aged 10–11 years. Most (89%) of the children aged 10–11 years were able to complete the child self-esteem measure, and 85 and 86% completed the peer and school connectedness measures, respectively.

### Clinical measures: dental caries experience and oral cleanliness

**Fig. 1:**
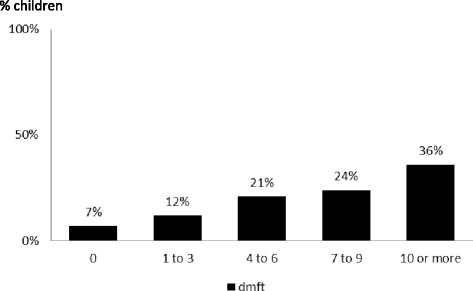
Severity of dental caries experience in Indonesian children aged 6–7 years

The children had experienced very high levels of dental caries (94% of the children aged 6–7 years and 90% of children aged 10–11 years) (Table 1). Not only was the prevalence of caries experience high with over 90% of the children aged 6–7 years having decayed teeth, but also the severity of their caries experience was considerable; in more than 80% of these children, 4 or more teeth were affected (Fig. 1). The impact of this level of untreated caries was high; 50% of the parents in both age groups reported that their child had experienced toothache in the previous 12 months. Substantial plaque was recorded on 49% of children aged 6–7 years, and 39% of those aged 10–11 years.

### Parental reporting of their children’s oral care habits

Tooth brushing was a commonly reported habit; only 10 parents reported that their children did not brush. The most common reported frequency was twice daily (78% of children aged 6–7 years and 75% of 10–11 years). However, the reported frequency of brushing was not consistent with the clinical examination of the plaque present on the children’s teeth. In 49% of children aged 6–7 years and in 38% of children aged 10–11 years, substantial amounts of visible plaque were present on at least one tooth surface. This indicates that, either brushing frequency was overestimated by the parents or, that tooth brushing was ineffectively performed by many of the children.

### Parental reporting on their child’s toothache experience

As noted, caries was very common in both age groups; 94% of children aged 6–7 years presented at least one decayed primary tooth, and 82% of children aged 10–11 years presented at least one decayed permanent tooth. Half of all children in both age groups were reported by their parents as having experienced toothache during the previous 12 months. Children experiencing toothache during the previous 12 months were significantly more likely to have caries than their peers without toothache (Table 2).

**Table 2:** Association between child’s dental caries and toothache during the previous 12 months (Chi square test)

	No toothache reported during the previous 12 months	Toothache reported during the previous 12 months	*p*-values
Decayed teeth (children aged 6–7 years)			
Yes (*n* = 379)	184 (48.6%)	195 (51.5%)	
No (*n* = 24)	21 (87.5%)	3 (12.5%)	*p* < 0.001**
Missing data (*n* = 136)			
Decayed permanent teeth (children aged 10–11 years)			
Yes (*n* = 240)	113 (47.1%)	127 (52.9%)	
No (*n* = 51)	34 (66.7%)	17 (33.3%)	*p* = 0.011*
Missing data (*n* = 154)			

*p < 0.05; **p < 0.005

### Children’s sugar consumption, child and parent toothbrushing behaviours and child oral cleanliness

The very high levels of dental caries found in the children studied here are likely to have occurred due to a combination of two key behaviors: very frequent sugar consumption and brushing with fluoride toothpaste less than twice a day. To obtain greater insight into these factors in this population, parents were asked about the nature and frequency of sugar intake by their children, the child’s use of toothbrush and toothpaste, and the parents’ own tooth-brushing behavior. Over 50% of children aged 6–7 years were reported by their parents to eat sugary foods between their meals, either every day (22%) or on most days (29%). Similar but lower levels (39%) were reported for children aged 10–11 years; sugary foods were consumed between meals either every day (14%) or on most days (25%). Soft drinks containing sugar were not the most common source of sweetened drinks consumed; two thirds of parents reported that their children drank soft drinks only occasionally (64% for children aged 6–7 years and 66% for children aged 10–11 years). Daily consumption of soft drinks containing sugar was reported to be relatively low at 9% for children aged 6–7 years and 3% for children aged 10–11 years. However, sugar was commonly added to sweeten drinks by 73% of parents of children aged 6–7 years and by 74% of parents of children aged 10–11 years. Among parents with children aged 6–7 years that sweetened drinks, 83% sweetened the drinks with sugar, 29% sweetened them with honey and 28% sweetened them with condensed milk. For children aged 10–11 years, the proportions were different; again, most parents sweetened the drinks with sugar (69%), but fewer used honey (13%) or condensed milk (18%).

The types of drinks being sweetened was of considerable concern; 31% of parents of children aged 6–7 years sweetened milk, 62% sweetened tea, and 2% sweetened water. Similar figures were found for parents of children aged 10–11 years; 24% sweetened milk, 67% sweetened tea, and 2% sweetened water. From a health promotion perspective, this implies that drinks that are often recommended as “healthy” (such as milk, tea and water) will become cariogenic as sugar has been added.

Although use of added sugar was widespread, brushing teeth with toothbrush and toothpaste was reported as a very common occurrence; only 1.4% of parents of children in both age groups never used toothpaste, and over 99% used a toothbrush. Thus, this prevention method is established; the remaining challenge is to ensure that teeth are brushed twice daily and effectively. Given the levels of children exhibiting substantial plaque on their teeth, it is possible that the parents over-reported their child’s brushing frequency. To gain insight into this possibility, parents were asked about their own frequency of tooth brushing. Asking about specific tooth brushing times (before or after breakfast, at bedtime, etc.) may reduce the likelihood of parents giving the socially desirable answer of “twice daily”. When the parent-reported data from the baseline questionnaires were analyzed in this way, approximately 1 in 3 (34%) parents reported brushing their own teeth only once a day.

### Association between toothache and school performance

As noted above, dental caries and reported toothbrushing were found to be almost universal, and toothache during the previous 12 months was found to be an important reflection of the impact of caries experience. Children aged 10–11 years who had experienced toothache were found to have significantly lower school performance than their peers (Table 3). One in three children were found to have excellent school performance; however, 11% of children aged 6–7 years and 6% of children aged 10–11 years underachieved, and 12% of children aged 6–7 years and 10% of children aged 10–11 years exhibited only fair school performance.

**Table 3:** Association between child’s oral cleanliness and school performance and between toothache during the previous 12 months and school performance (Chi square test)

	Under-achieving	Fair	Good	Excellent	*p*-values
Substantial plaque. Children aged 6–7 years					
Yes (*n* = 233)	23 (9.8%)	35 (15.0%)	109 (46.8%)	66 (28.3%)	*p* = 0.032*
No (*n* = 244)	24 (9.8%)	20 (8.2%)	106 (43.4%)	94 (38.5%)	
Missing data (*n* = 62)					
Children aged 10–11 years					
Yes (*n* = 152)	10 (6.6%)	22 (14.5%)	82 (54.0%)	38 (25.0%)	*p* = 0.002**
No (*n* = 239)	10 (4.2%)	18 (7.5%)	110 (46.0%)	101 (42.3%)	
Missing data (*n* = 54)					
Reported toothache during the previous 12 months. Children aged 6–7 years					
Yes (*n* = 215)	19 (8.8%)	34 (15.8%)	97 (45.1%)	65 (30.2%)	*p* = 0.089
No (*n* = 220)	23 (10.5%)	18 (8.2%)	101 (45.9%)	78 (35.5%)	
Missing data (*n* = 104)					
Children aged 10–11 years					
Yes (*n* = 155)	4 (2.6%)	18 (11.6%)	90 (58.1%)	43 (27.4%)	*p* = 0.042*
No (*n* = 154)	7 (4.6%)	14 (9.1%)	69 (44.8%)	64 (41.6%)	
Missing data (*n* = 136)					

*p < 0.05; **p < 0.005

### Association between oral cleanliness and school performance

The presence of substantial plaque on teeth results from infrequent or inadequate tooth brushing. The presence of substantial plaque was found to have a significant association with child’s school performance at both age groups; 6–7 years and 10–11 years (Table 3).

### Associations between self-esteem and oral health

Self-esteem was measured through self-reports by children aged 10–11 years using a validated measure. As might be expected, child’s self-esteem was significantly associated with child’s school performance, such that significantly more children that self-rated as having higher levels of self-esteem had good or excellent school performance. However, no significant associations were found between child’s self-esteem and toothache or caries experience.

## Discussion

Dental caries was found to occur at very high levels in children attending public schools in Indonesia. This was found to be associated with high sugar consumption and inadequate tooth brushing, which together cause substantial dental plaque. Preventive programs should advise sugar restriction [22] and brushing of the teeth with fluoride toothpaste twice daily. Dental diseases are prevalent globally and, despite great improvement in their prevention and treatment, issues such as pain, anxiety, functional limitations (including poor school performance) and social handicaps (due to tooth loss) remain. The treatment of dental disease is expensive, consuming 5–10% of health care budgets in industrialized countries, and, if the same treatment approaches were used, could consume the entire financial resources available for health care in most low-income countries. Therefore, taking a preventive approach to enhance health provides an economically sound argument. For both adults and children, the World Health Organization recommends limiting sugar intake to less than 10% of total energy intake [22]. A small reduction in the risk of dental caries in childhood is of significance in later life [23]. School settings provide an ideal opportunity to reinforce prevention routines and, where these routines have not yet been established at home, schools represent an excellent opportunity to support children and families to establish the routine of twice daily tooth brushing [24]. Although the schools studied here are typical of the Indonesian public elementary system, they are not necessarily representative nationally. This study is cross-sectional, and the conclusions that can be drawn relate only to associations between the studied factors.

Based on the results of the dental visual impact assessments, teachers were clearly able to identify a child as having decayed teeth based on their smile, and the vast majority viewed that child as unhealthy. Most of the teachers judged that a dentally unhealthy appearance (e.g., decayed front teeth), even in children, could adversely impact their future employment prospects. Caries can affect child health in a variety of ways, including toothache, which is associated with lower school performance. Although the reason for absence was not specified in the school records, given the high occurrence of caries experience and associated morbidity, it is likely some days were lost due to the impact of poor oral health [6, 7]. Caries in permanent incisors can markedly affect smile and self-image. Moreover, parents’ own self-esteem can significantly affect their child’s self-esteem during life transitions [25]. The critical role of parents and teachers in influencing change is key to enhancing the tooth brushing behaviors of children aged 6–7 years and 10–11 years. These two age groups were selected because they represent important life transitions; at 6–7 years, the first permanent molars are erupting; if these teeth can be prevented from developing dental caries, lifelong benefits can ensue. From a child-development perspective, anterior deciduous teeth begin to shed at this age, and self-awareness emerges. At 10–11 years, children are completing their primary education and stand on the threshold of the transition to senior school, a time of considerable personal challenge and self-identity within a new environment [26].

The dental visual impact study results showed that children with good oral health were perceived by teachers to be more likely to have good employment prospects. Visual screening performed by teachers in detection of dental caries has been shown to correlate with clinical dental examination [27]. Previous findings show that caries in the primary dentition are predictive of caries in permanent teeth [28]. Socio economic status (SES), as measured by educational attainment and employment type is associated with oral health of young adults and over the life course. The health selection hypothesis proposes that childhood health is linked to adult SES and SES gradients in adult health through potential health selection and social causation effects [29]. In short, this cross-sectional study does not verify a cause-and-effect relationship between poor oral health, poor school performance, and perceived employment prospects. The study demonstrates an association which can be investigated further to determine whether improving children’s oral health improves their school performance and perceived future employment prospects.

This study was conducted in Indonesia, to explore child oral health and to assess the impact of child oral health on self- esteem, school performance and perceived employability. The cross- sectional study design limits analysis to associations between variables but provides an important starting point for a longitudinal cohort study. Most of the variables, except oral health status measured by clinical examination, were through questionnaires and self-reported measures, introducing the possibility of information bias, and bias due to social desirability. Any study attempting to measure reported behavior and attitudes must consider the extent to which socially acceptable answers could impact on the interpretation of the results [18]. The visual dental impact measure could also have been influenced by the teachers’ own perceptions. Although not a nationally representative sample, the population selected typically represents Indonesian public elementary schools. The study is limited to examining associations only, due to the cross-sectional study design and a future prospective cohort study could explore causation. Overall the research is the first of its kind in Indonesia and throws light on the patterns of sugar consumption and its effect on oral health and future life prospects. It also highlights the need to take necessary preventive measures to address these issues.

Important oral health and related behavioral data are provided for the first time for children in disadvantaged communities in Indonesia. Longer follow-up times and additional studies are warranted to determine the impact of enhancing oral health on children’s academic performance in school. Understanding how individual behaviors within the family, community and broader society can affect health is critical for enhancing prevention [30, 31]. Repairing the damage caused by dental caries is difficult and costly; therefore, the vast majority of children’s caries remains untreated in Indonesia [32]. As secondary prevention and curative care are inefficient and expensive, the primary prevention of dental caries remains a key objective for Indonesia.

## Conclusion

Dental caries is common among Indonesian children due to the frequent use of added sugar and inadequate tooth brushing with fluoride toothpaste. Caries has a wide variety of impacts on child health, including toothache which is associated with lower self-esteem and poorer school performance. Preventive programs should advise sugar restriction, particularly addressing the practice of adding sugar to milk and water. As tooth brushing with toothpaste is an established social norm that has the potential to improve oral health in Indonesia, school programs that increase tooth-brushing frequency with fluoride toothpaste to twice daily can also be recommended.

## Additional files

Additional file 1: Appendix 1. The dental visual impact study form. (PDF 219 kb)

Additional file 2: Appendix 2. The Child reported brushing measure. (PDF 196 kb)

Additional file 3: Appendix 3. The child self esteem measure. (PDF 281 kb)

Additional file 4: Appendix 4. The oral health behaviors questionnaire to parents. (PDF 403 kb)
