# Supplementary material for: An assessment of the impacts of child oral health in Indonesia and associations with self-esteem, school performance and perceived employability
Source: BMC Oral Health. 2017 Mar 21;17:65. doi: 10.1186/s12903-017-0358-5 (PMC5361816; doi:10.1186/s12903-017-0358-5)
Supplement: Supplementary file 1 — Appendix 1. The dental visual impact study form. (PDF 219 kb) [file 12903_2017_358_MOESM1_ESM.pdf]

Aditional file 1

Dental Visual Impact Study
(Scale author: Professor Cynthia Pine)

Dental expert to show slides to teachers as part of Teachers' Workshop in both Test and Control groups at the beginning of the study. Invite all teachers in school to attend the Workshop. Explain schools are involved in a dental research study and that it is very important that we understand the teachers' perspective and that we will be seeking their views as part of this study. As part of that, they first complete the WHO Teachers' Questionnaire. Next, we want to understand your views on the following pictures of child oral health. Each slide will be shown for 30 seconds, at the end of this time you will be asked to score each slide for a number of characteristics. Dental expert will show 4 slides of child smiles (not full faces) showing teeth with (2 slides) and without (2 slides) caries experience and poor/good gingival health.

Teachers' score sheets for dental slides: Please tick √ the answer that you consider the most appropriate. There are no right or wrong answers, we just want to understand your views.

Show Slide 1

In your opinion……

1. Do you consider this smile to be of a healthy child or an unhealthy child?

Answer: ☐Healthy ☐Unhealthy ☐Don't know

2. Is this child more or less likely to be absent from school more often than their peers?

Answer: ☐More likely ☐Less likely ☐Don't know

3. Is this child more or less likely to do well academically at school?

Answer: ☐More likely ☐Less likely ☐Don't know

4. Is this child more or less likely to be socially excluded from their peers?

Answer: ☐More likely ☐Less likely ☐Don't know

5. Is this child more likely or less likely to have good employment prospects in the future?

Answer: ☐More likely ☐Less likely ☐Don't know

Show Slide 2

In your opinion…… Repeat Response Sheet.
