# Supplementary material for: An assessment of the impacts of child oral health in Indonesia and associations with self-esteem, school performance and perceived employability
Source: BMC Oral Health. 2017 Mar 21;17:65. doi: 10.1186/s12903-017-0358-5 (PMC5361816; doi:10.1186/s12903-017-0358-5)
Supplement: Supplementary file 2 — Appendix 2. The Child reported brushing measure. (PDF 196 kb) [file 12903_2017_358_MOESM2_ESM.pdf]

Child ID Number…………

Additional file 2

Child Reported Brushing Measure 6-7 years olds
(Scale author: Professor Cynthia Pine)

Instruction to Teacher: Please ensure that each child answers the questions separately and that it is an individual child response, not from a group or copied from their friends.

Tell me about this morning at home,

Did you wash your hands?	YES	NO
Did you wash your face?	YES	NO
Did you brush your teeth?	YES	NO
Did you have any breakfast?	YES	NO

Tell me about before you went to bed yesterday,

Did you wash your hands?	YES	NO
Did you wash your face?	YES	NO
Did you brush your teeth?	YES	NO

Child ID Number…………

Child Reported Brushing Measure 10-11 year olds
(Scale author: Professor Cynthia Pine)

Instruction to Teacher: Please ensure that each child answers the questions separately and that it is an individual child response, not from a group or copied from their friends.

Tell me about this morning at home,

Did you wash your hands?	YES	NO
Did you wash your face?	YES	NO
Did you brush your teeth?	YES	NO
Did you have any breakfast?	YES	NO

Tell me about before you went to bed yesterday

Did you wash your hands?	YES	NO
Did you wash your face?	YES	NO
Did you brush your teeth?	YES	NO

There are no right or wrong answers, please choose the answer you think is the closest to how you feel.

SD = strongly disagree, **D** = disagree, **A** = agree, **SA** = strongly agree

Sometimes I forget to brush my teeth in the morning	SD	D	A	SA
I don't need to brush my teeth at bedtime	SD	D	A	SA
If I brush my teeth in the morning, I will have a healthy smile	SD	D	A	SA
I think it is important to brush my teeth in the morning and at bedtime	SD	D	A	SA
I plan to brush my teeth every day	SD	D	A	SA
My friends think it is important to brush teeth in the morning and at bedtime	SD	D	A	SA
Sometimes I forget to brush my teeth at bedtime	SD	D	A	SA
If I brush my teeth in the morning and at bedtime, I will have a healthy smile	SD	D	A	SA
In my family, we only brush our teeth in the morning	SD	D	A	SA
