# Supplementary material for: An assessment of the impacts of child oral health in Indonesia and associations with self-esteem, school performance and perceived employability
Source: BMC Oral Health. 2017 Mar 21;17:65. doi: 10.1186/s12903-017-0358-5 (PMC5361816; doi:10.1186/s12903-017-0358-5)
Supplement: Supplementary file 3 — Appendix 3. The child self esteem measure. (PDF 281 kb) [file 12903_2017_358_MOESM3_ESM.pdf]

Child ID Number: …………

Additional file 3

Self Esteem Measure for 6-7 year olds

Instruction to Teacher: Please read the statements with the child. Help the child to complete if needed. But answers are to be from the child. Make it a fun game.

……………………………………………………………………………………………

Put a cross on the line to the closest to how you feel.

I like myself ………………………….

I feel there are many good things about me. ……………………………

I feel proud of myself. …………………………..

I feel confident. ………………………..

I like the way I am. ……………………….

Child ID Number: …………

Self Esteem Measure for 10-11 year olds
(With sub scales of peer and school connectedness)

There are no right or wrong answers, please choose the answer you think is the closest to how you feel.

SD = strongly disagree, **D** = disagree, **A** = agree, **SA** = strongly agree

I like myself.	SD	D	A	SA
I feel there are many good things about me.	SD	D	A	SA
I feel proud of myself.	SD	D	A	SA
I feel confident.	SD	D	A	SA
I like the way I am	SD	D	A	SA
I have as many friends as others my age.	SD	D	A	SA
I am as popular as others my age.	SD	D	A	SA
Other people my age think I am fun to be with.	SD	D	A	SA
Other people my age wish they were like me.	SD	D	A	SA
I am a person others turn to for help.	SD	D	A	SA
My teachers are fair with what they expect from me.	SD	D	A	SA
I am usually proud of my report card.	SD	D	A	SA
School is as hard for me as it is for most others.	SD	D	A	SA
My teachers are happy with the kind of work I do.	SD	D	A	SA
Most of my teachers understand me.	SD	D	A	SA
