# Supplementary material for: An assessment of the impacts of child oral health in Indonesia and associations with self-esteem, school performance and perceived employability
Source: BMC Oral Health. 2017 Mar 21;17:65. doi: 10.1186/s12903-017-0358-5 (PMC5361816; doi:10.1186/s12903-017-0358-5)
Supplement: Supplementary file 4 — Appendix 4. The oral health behaviors questionnaire to parents. (PDF 403 kb) [file 12903_2017_358_MOESM4_ESM.pdf]

Additional file 4

Oral Health Behaviours Questionnaire to Parents
(Scale author: Adair et al, 2004)

Thank you for agreeing to take part in this dental study designed to give us an understanding of why some children develop tooth decay. We are trying to understand the wide range of dental beliefs and behaviours that families have and develop about their children's teeth. In this questionnaire there are no right or wrong answers – we are just trying to understand what is usual for your family.

This questionnaire is divided into sections. The wording of some questions may seem a little unusual to you because it has been designed to be completed in many different countries with different ways of life.

All information given in this questionnaire will be treated confidentially.

General Information

We would like you to complete this questionnaire by providing us with information about your child.

Child's name ……………………………………………….

Child's date of birth ____dd / ____ mm / ____ yy

Child's gender: male ☐1 female ☐2

The following questions are about the above named child.

Section A

The first set of questions are about visiting the dentist, toothache, and general questions about your child's baby teeth and dental health.

1. **Before today, have you ever taken your child to a dentist?** Yes ☐1 No ☐2
   If yes, did the dentist examine your child's teeth? Yes ☐1 No ☐2

2. **Has your child ever had toothache in the last year?** Yes ☐1 No ☐2
   If yes, how often? once ☐1 twice ☐2
   three times ☐3 more ☐4

3. **If your child gets toothache would you:**

give your child painkillers	☐1	obtain antibiotics	☐2
go to the dentist	☐3	go to the doctor	☐4
use a herbal remedy	☐5	ask for the tooth to be taken out	☐6
do nothing, it will get better on its own	☐7	consult family	☐8
go to chemist	☐9	seek other medical care	☐10

The next set of questions examine feelings and attitudes towards tooth decay and toothbrushing. ***Please tick one box on each line.***

	strongly disagree	disagree	neither agree or disagree	agree	strongly agree
4. As a family, we are confident that we can reduce the chances of our child getting tooth decay	1	2	3	4	5
5. Tooth decay will not get better by itself	1	2	3	4	5
6. Regular visits to the dentist would be effective in stopping our child having tooth decay	1	2	3	4	5
7. Tooth decay would have major consequences on our child’s general health	1	2	3	4	5
8. Tooth decay is a serious problem in baby teeth	1	2	3	4	5
9. As parents, it is our responsibility to prevent our child getting tooth decay.	1	2	3	4	5
10. Our child losing a baby tooth due to tooth decay would be upsetting	1	2	3	4	5
11. We feel it is important that we check our child’s teeth for decay	1	2	3	4	5
12. If our child does not want to brush his/her teeth every day we don’t feel we should make them	1	2	3	4	5
13. It is important to clean my child’s teeth every day so my child has a nice smile	1	2	3	4	5
14. It is the responsibility of the dentist to prevent our child getting tooth decay	1	2	3	4	5
15. No matter what we do, our child is likely to get tooth decay	1	2	3	4	5
16. We can prevent tooth decay in our child by reducing sugary foods and drinks between meals	1	2	3	4	5
17. It is just bad luck if our child gets tooth decay	1	2	3	4	5
18. As a family we intend brushing our child’s teeth for him/her	1	2	3	4	5
19. We intend brushing our child’s teeth for him/her twice a day	1	2	3	4	5

	**strongly disagree**	**disagree**	**neither agree or disagree**	**agree**	**strongly agree**
20. The people in my family would feel it was important to help brush our child's teeth twice a day	1	2	3	4	5
21. The people we know well would feel it was important to brush our child's teeth twice a day	1	2	3	4	5
22. We feel able to brush our child's teeth for him/her	1	2	3	4	5
23. I don't know how to brush my child's teeth properly	1	2	3	4	5
24. If we brush our child's teeth twice a day, we can prevent our child getting tooth decay in the future	1	2	3	4	5
25. If our child uses a fluoride toothpaste, it will prevent tooth decay	1	2	3	4	5
26. A toothpaste without fluoride will prevent tooth decay	1	2	3	4	5
27. We can prevent tooth decay in our child by helping with brushing once a day	1	2	3	4	5
28. Our child not toothbrushing once a day would cause tooth decay	1	2	3	4	5
29. If our child gets tooth decay, it is by chance.	1	2	3	4	5
30. It would not make any difference to our child getting tooth decay, if we helped him/her brush every day	1	2	3	4	5
31. We feel it is important to check *if* our child has brushed his/her teeth	1	2	3	4	5
32. We don´t have time to help brush our child´s teeth twice a day	1	2	3	4	5
33. We cannot make our child brush his/her teeth twice a day	1	2	3	4	5
34. My child's teeth are brushed as part of my child's daily washing routine (washing hands and face)	1	2	3	4	5
35. Buying toothbrushes and toothpaste for the whole family is expensive.	1	2	3	4	5
36. Tooth decay runs in families.	1	2	3	4	5
37. Some people just naturally have soft teeth.	1	2	3	4	5

Section B

The next set of questions are about toothbrushing / tooth cleaning.

1. What is used to clean your child's teeth? ***(Please tick as many boxes as necessary)***

Toothbrush ☐1 Chewing sticks ☐2

Cloth ☐3 Finger ☐4

Other ☐5 (please specify) ……………………………………………………

Nothing used ☐6

What else do you use?

Toothpaste ☐1 Salt ☐2

Powder ☐3 Other ☐4 (please specify) …………………………

Nothing ☐5

2. Who brushes/cleans your child's teeth? ***(Please tick as many boxes as necessary)***

child ☐1 parent ☐2

someone else ☐3 teeth are not brushed / cleaned ☐4

3. How often are your child's teeth brushed/cleaned? ***(Please tick one box)***

Never ☐1 Not every day ☐2

Once a day ☐3 Twice a day ☐4

Three times a day ☐5 Every other day ☐6

4. How old was your child when he/she first started having his/her teeth brushed/cleaned?

Under 1 year ☐1 1 year – under 2 years ☐2

2 years – under 3 years ☐3 3 years or over ☐4

Cannot remember ☐5 Teeth are not brushed/cleaned ☐6

5. How old was your child when he/she started brushing/cleaning his/her teeth on his/her own?

Under 2 years ☐1 2 years – under 3 years ☐2

3 years or over ☐3 Cannot remember ☐4

Does not brush / clean their teeth ☐5

6. Has your child always brushed/cleaned his/her own teeth?

Yes ☐1

No, someone used to help ☐2

Does not brush / clean their teeth ☐3

Please tick one box on each line.

	**Every day**	**Most days**	**Occasionally**	**Never**
7. Do you remind your child to brush/clean their teeth?	1	2	3	4
8. Do you check they have brushed/cleaned their teeth?	1	2	3	4
9. Do you watch them while they brush/clean their teeth?	1	2	3	4
10. Do you help them to brush/clean their teeth?	1	2	3	4
11. Do you brush/clean their teeth for them?	1	2	3	4

12. When do you brush/clean your child's teeth? ***(Please tick as many boxes as necessary)***

When they first get up in the morning ☐1 After breakfast ☐2

Before midday meal ☐3 After midday meal ☐4

Before evening meal ☐5 After evening meal ☐6

Before going to sleep at night ☐7 Teeth are not brushed ☐8

Other occasions ☐9 please specify …………………………………….

13. When does your child brush/clean his/her teeth by themself? ***(Please tick as many boxes as necessary)***

When they first get up in the morning ☐1 After breakfast ☐2

Before midday meal ☐3 After midday meal ☐4

Before evening meal ☐5 After evening meal ☐6

Before going to sleep at night ☐7 Child does not brush their own teeth ☐8

Other occasions ☐9 please specify ………………………………………

14. People start using toothpaste at different ages. Has your child started using toothpaste?

Yes, always ☐1 Yes, sometimes ☐2 No ☐3

If yes, which brand of toothpaste do you <u>usually</u> buy for your child to use?

Brand name ……………….…………………………..

How old was your child when you first used toothpaste for him/her?

Under 1 year ☐1 | 1 year – under 2 years ☐2

2 years – under 3 years ☐3 | 3 years or over ☐4

Cannot remember ☐5 | Does not use toothpaste ☐6

15. When your child's teeth are brushed, do you use toothpaste or not?

Never use toothpaste ☐1 | Sometimes use toothpaste ☐2

Always use toothpaste ☐3

16. Does your child use a toothbrush? Yes ☐1 No ☐2

If yes, does your child:

have his/her own toothbrush? ☐1

OR do they share a toothbrush with someone else? ☐2

17. If your child uses toothpaste, how much toothpaste does your child usually use on his/her toothbrush? Please tick the picture which most closely resembles the amount of toothpaste you use.

1 ☐ 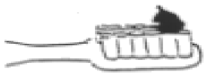

2 ☐ 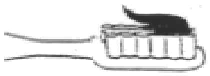

3 ☐ 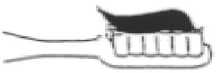

<u>Section C</u>

The following questions are related to eating and drinking.

1. When your child was a baby, did you breast or bottle feed?

breast feed ☐1

bottle feed (with formula milk) ☐2

breast and bottle feed ☐3

(Questions 2-6 have been removed as they are for families of younger children.)

7. **Have you ever had advice about what your child should or should not be eating or drinking to look after his/her teeth?**

Yes ☐1 No ☐2

If yes, who has advised you?

family ☐1 friends ☐2 dentist ☐3 doctor ☐4 health visitor ☐5

baby clinic ☐6 other ☐7 please specify …………………………………

Please tick one box on each line.

	Every day	Most days	Once a week	Occasionally	Never
8. How often does your child eat sweets (including chocolates)?	1	2	3	4	5
9. How often does your child eat sugary foods between meals (for example, biscuits, cake, jam)?	1	2	3	4	5
10. How often does your child drink soft drinks containing sugar? (including squash, fizzy drinks, etc; not "diet" type drinks)	1	2	3	4	5

11. **What does your child usually eat/drink within an hour before going to bed to sleep at night?**

Eats 1 ……………………………………………..

Drinks 2 ……………………………………………..

Does not eat/drink before going to bed ☐3

12. **Many children take a drink to bed with them either to have before they go to sleep, or during the night. How often does your child have something to drink in bed or during the night? *(Please tick one box)***

Every day ☐1 Most days ☐2

Occasionally ☐3 Never ☐4

13. When your child has a drink in bed or during the night, what does he/she usually have? *(Please tick as many boxes as necessary)*

Milk ☐1 Milk drinks (eg. chocolate milk) ☐2

Milk with sugar or honey ☐3 Fruit juices ☐4

Fruit squashes ☐5 Fizzy drinks ☐6

Tea/coffee ☐7 Water ☐8

Herbal drinks/tea ☐9 Other ☐10 (please specify)…………………

Never has a drink in bed ☐11

14. Thinking about food, how often does your child eat in bed or during the night? *(Please tick one box)*

Every day ☐1 Most days ☐2 Occasionally ☐3 Never ☐4

15. When your child has something to eat when going to sleep or during the night, what does he/she usually have?

Sweet biscuits (including chocolate biscuits) ☐1 Fruit ☐2

Savoury and plain biscuits (including cheese biscuits ☐3 Sandwiches (sweet) ☐4

Cakes ☐5 Sweets or chocolate ☐6

Crisps or savoury snacks ☐7 Never eats in bed ☐8

Other ☐9 please specify ………………

16. When you sweeten your child's drinks, what do you add?

Sugar ☐1 Honey ☐2

Condensed milk ☐3 Never sweeten child's drinks ☐4

Other ☐5 please specify ………………………………

17. Which drinks do you sweeten?

Milk ☐1 Water ☐2

Tea ☐3 Other ☐4 please specify ………………….

The next set of questions examine feelings and attitudes towards sugary foods and drinks.
Please tick one box on each line

	strongly disagree	disagree	neither agree or disagree	agree	strongly agree
18. As a family, we intend controlling how often our child has sugary foods or drinks between meals	1	2	3	4	5
19. The people in my family would feel it was important to control how often our child has sugary foods and drinks between meals	1	2	3	4	5
20. As a family, we feel it is difficult for us to stop our child having sugary foods and drinks between meals	1	2	3	4	5
21. We feel able to give our child healthy alternatives to sugary foods between meals (eg. like apples instead of sweets)	1	2	3	4	5
22. We feel able to give our child healthy alternatives to sugary drinks between meals (eg. like water instead of a fizzy drink)	1	2	3	4	5
23. It is worthwhile to give our child sweets/biscuits to behave well.	1	2	3	4	5
24. Our child eating sugary foods and drinks in between meals would cause tooth decay	1	2	3	4	5
25. The people we know well would feel it was important to control how often our child has sugary foods and drinks	1	2	3	4	5
26. In our family, it would be unfair not to give sweets to our child every day	1	2	3	4	5
27. It is often too stressful to say no to my child when they want sweets	1	2	3	4	5
28. When our child is tired, it can be a struggle to brush his/her teeth	1	2	3	4	5
29. Bringing our child to the dentist on a regular basis is the best way to prevent tooth decay	1	2	3	4	5
30. It is not worth it to battle with our child to brush his/her teeth twice a day	1	2	3	4	5
31. It is just bad luck if our child gets tooth decay	1	2	3	4	5
32. The dentist is the best person to prevent tooth decay in our child	1	2	3	4	5

<u>Section D</u>

The following questions are related to <u>your</u> experiences of visiting the dentist and oral care

1. What is your usual reason for going to see a dentist? ***(Please tick one box)***

Regularly for a check up ☐ 1

Regularly for treatment ☐ 2

Only if I have problems with my teeth or gums ☐ 3

I do not visit a dentist ☐ 4

2. What brand of toothpaste do you <u>usually</u> use? ……………………………………

3. When do you brush your teeth? ***(Please tick as many boxes as necessary)***

When you first get up in the morning ☐ 1 After breakfast ☐ 2

Before mid-day meal ☐ 3 After mid-day meal ☐ 4

Before evening meal ☐ 5 After evening meal ☐ 6

Before going to bed ☐ 7 Do not brush every day ☐ 8

Other occasions ☐ 9 please specify …………………………………………….

Please tick one box on each line.

	**Every day**	**Most days**	**Occasionally**	**Never**
How often do you use the following?				
4. Dental floss	1	2	3	4
5. Mouthrinses	1	2	3	4
6. Sugar-free chewing gum	1	2	3	4

<u>Section E</u>

<u>Now to the final questions</u>. People have different care arrangements for their children. The following questions help us understand child care routines, and the section ends with a few routine questions on background information.

1. **Who usually looks after your child during the day? (*Please tick one box*)**

   Mother at home ☐1 Father at home ☐2

   Sister/brother ☐3 Child's grandparent ☐4

   Other relative ☐5 Friend/neighbour ☐6

   Paid childminder ☐7 Other ☐11 …………………………

2. **Does your child sleep overnight at the school or childminder?**

   Never ☐1 Occasionally ☐2

   Regularly each month ☐3 Regularly each week ☐4

3. **Who does your child live with? (*Tick as many boxes that apply*)**

   Mother ☐1 Father ☐2

   Mother and father ☐3 Mother and stepfather ☐4

   Father and stepmother ☐5 Grandparents ☐6

   Other relatives ☐7 please specify …………………………………….

   Other ☐8 please specify …………………………………….

4. **How many children are living in your house now?** ……………

5. **Is this your first child, second child etc?** ………………..

6. **Are you the child's:** mother ☐1 father ☐2 other ☐3 please specify ……….

7. **What is your age?** Under 20☐1 20 - 30☐2 31 - 40☐3 over 40 ☐4

8. **What is your marital status?** Married ☐1 Single ☐2

   Divorced / separated? ☐3 Widowed ☐4

9. **What is your occupation?** ……………………………………………………………..

10. **What is the postcode of your home address?** ……………………………..…………

11. At what level did the child's mother finish her full-time education?

Primary school	☐1	Secondary school	☐2
Further education (college)	☐3	Higher education (university)	☐4
No formal education	☐5		
Other	☐6 please specify ……………………………		

12. At what level did the child's father finish his full-time education?

Primary school	☐1	Secondary school	☐2
Further education (college)	☐3	Higher education (university)	☐4
No formal education	☐5	Other	☐6 please specify ...…………………

Please take a moment to ensure that you have answered all the questions.

Thank you very much for your help.

Please return this completed questionnaire to:

Your child's class teacher
